# A geospatial evaluation of timely access to surgical care in seven countries

**DOI:** 10.2471/BLT.16.175885

**Published:** 2017-03-16

**Authors:** Lisa M Knowlton, Paulin Banguti, Smita Chackungal, Traychit Chanthasiri, Tiffany E Chao, Bernice Dahn, Milliard Derbew, Debashish Dhar, Micaela M Esquivel, Faye Evans, Simon Hendel, Drake G LeBrun, Michelle Notrica, Iracema Saavedra-Pozo, Ross Shockley, Tarsicio Uribe-Leitz, Boualy Vannavong, Kelly A McQueen, David A Spain, Thomas G Weiser

**Affiliations:** a Department of Surgery, Division of General Surgery, Stanford University School of Medicine, 300 Pasteur Drive, S067, Stanford, CA 94305, United States of America (USA).; b Department of Anaesthesia, University of Rwanda, Butare, Rwanda.; c London Health Sciences Centre, London, Canada.; d Department of Anaesthesia, Mahosot Hospital, Vientiane, Lao People's Democratic Republic.; e Department of Surgery, Massachusetts General Hospital, Boston, USA.; f Ministry of Health and Social Welfare, Monrovia, Liberia.; g School of Medicine, Addis Ababa University, Addis Ababa, Ethiopia.; h National Institute of Diseases of Chest and Hospital, Dhaka, Bangladesh.; i Department of Anesthesiology, Boston Children’s Hospital, Boston, USA.; j Department of Anaesthesiology, The Alfred Hospital, Melbourne, Australia.; k Perelman School of Medicine, University of Pennsylvania, Philadelphia, USA.; l Global Surgical Consortium, Nashville, USA.; m Department of Surgery, Caja Nacional de Salud Hospital, La Paz, Plurinational State of Bolivia.; n Department of Otolaryngology, Vanderbilt University Medical Center, Nashville, USA.; o Department of Anesthesiology, Vanderbilt University Medical Center, Nashville, USA.

## Abstract

**Objective:**

To assess the consistent availability of basic surgical resources at selected facilities in seven countries.

**Methods:**

In 2010–2014, we used a situational analysis tool to collect data at district and regional hospitals in Bangladesh (*n* = 14), the Plurinational State of Bolivia (*n* = 18), Ethiopia (*n* = 19), Guatemala (*n* = 20), the Lao People's Democratic Republic (*n* = 12), Liberia (*n* = 12) and Rwanda (*n* = 25). Hospital sites were selected by pragmatic sampling. Data were geocoded and then analysed using an online data visualization platform. Each hospital’s catchment population was defined as the people who could reach the hospital via a vehicle trip of no more than two hours. A hospital was only considered to show consistent availability of basic surgical resources if clean water, electricity, essential medications including intravenous fluids and at least one anaesthetic, analgesic and antibiotic, a functional pulse oximeter, a functional sterilizer, oxygen and providers accredited to perform surgery and anaesthesia were always available.

**Findings:**

Only 41 (34.2%) of the 120 study hospitals met the criteria for the provision of consistent basic surgical services. The combined catchments of the study hospitals in each study country varied between 3.3 million people in Liberia and 151.3 million people in Bangladesh. However, the combined catchments of the study hospitals in each study country that met the criteria for the provision of consistent basic surgical services were substantially smaller and varied between 1.3 million in Liberia and 79.2 million in Bangladesh.

**Conclusion:**

Many study facilities were deficient in the basic infrastructure necessary for providing basic surgical care on a consistent basis.

## Résumé

**Objectif:**

Évaluer la disponibilité constante des ressources chirurgicales de base dans certains établissements de sept pays.

**Méthodes:**

En 2010–2014, nous avons utilisé un outil d'analyse de situation pour recueillir des données dans des hôpitaux régionaux et de district au Bangladesh (*n* = 14), dans l'État plurinational de Bolivie (*n* = 18), en Éthiopie (*n* = 19), au Guatemala (*n* = 20), au Liberia (*n* = 12), dans la République démocratique populaire lao (*n* = 12) et au Rwanda (*n* = 25). Les sites ont été sélectionnés par échantillonnage pragmatique. Les données ont été géocodées puis analysées à l'aide d'une plate-forme de visualisation des données en ligne. La population desservie par chaque hôpital a été définie comme celle pouvant s'y rendre à bord d'un véhicule en deux heures maximum. Nous avons considéré qu'un hôpital disposait en permanence des ressources chirurgicales de base si celui-ci avait toujours à disposition de l'eau claire, de l'électricité, des médicaments essentiels, notamment des solutions intraveineuses, et au moins un anesthésique, un analgésique, un antibiotique, un oxymètre de pouls fonctionnel, un stérilisateur fonctionnel, de l'oxygène et des prestataires accrédités pour effectuer des interventions chirurgicales et des anesthésies.

**Résultats:**

Seuls 41 (34,2%) des 120 hôpitaux analysés remplissaient les critères définissant une offre constante de services chirurgicaux de base. La population totale desservie par les hôpitaux analysés dans chaque pays allait de 3,3 millions de personnes au Liberia à 151,3 millions de personnes au Bangladesh. Cependant, la population totale desservie par les hôpitaux analysés dans chaque pays et qui remplissaient les critères définissant une offre constante de services chirurgicaux de base était nettement inférieure, allant de 1,3 millions de personnes au Liberia à 79,2 millions de personnes au Bangladesh.

**Conclusion:**

De nombreux établissements analysés n'avaient pas les infrastructures élémentaires nécessaires pour offrir de façon constante des soins chirurgicaux de base.

## Resumen

**Objetivo:**

Evaluar la disponibilidad coherente de recursos quirúrgicos básicos en centros seleccionados de siete países.

**Métodos:**

En 2010–2014, se utilizó una herramienta de análisis situacional para recopilar datos en hospitales de distrito y regionales de Bangladesh (*n* = 14), el Estado Plurinacional de Bolivia (*n* = 18), Etiopía (*n* = 19), Guatemala (*n* = 20), la República Democrática Popular Lao (*n* = 12), Liberia (*n* = 12) y Rwanda (*n* = 25). Los hospitales se seleccionaron según un muestreo pragmático. Los datos se geocodificaron y posteriormente se analizaron utilizando una plataforma de visualización de datos en línea. La población beneficiaria de cada hospital se definió como aquellas personas que podían llegar al hospital con un viaje en vehículo de dos horas como máximo. Solo se consideraba que un hospital mostraba una disponibilidad coherente de recursos quirúrgicos básicos si contaba en todo momento con agua potable, electricidad, medicamentos esenciales, incluidos fluidos intravenosos y, al menos, un anestésico, un analgésico y un antibiótico, un oxímetro de pulso funcional, un esterilizador funcional, oxígeno y proveedores acreditados para realizar cirugía y administrar anestesia.

**Resultados:**

Únicamente 41 (34,2%) de los 120 hospitales del estudio cumplieron con los requisitos de suministro constante de servicios quirúrgicos básicos. Los beneficiarios combinados de los hospitales del estudio en cada país de estudio oscilaron entre 3,3 millones de personas en Liberia y 151,3 millones de personas en Bangladesh. Sin embargo, los beneficiarios combinados de los hospitales del estudio en cada país de estudio que cumplieron los criterios de suministro constante de servicios quirúrgicos básicos fueron mucho menores y oscilaron entre 1,3 millones en Liberia y 79,2 millones en Bangladesh.

**Conclusión:**

Muchos de los centros del estudio carecían de la infraestructura básica necesaria para suministrar atención quirúrgica básica de forma coherente.

## ملخص

الغرض تقييم التوافر المستمر للموارد الجراحية الأساسية في المرافق المحددة في سبع دول.

الطريقة في الفترة الممتدة ما بين عاميّ 2010 وحتى 2014، استخدمنا أداة تحليل ظرفية لجمع البيانات في المستشفيات الواقعة في الأحياء والمناطق في بنغلاديش (عدد * = 14 *)، ودولة بوليفيا متعددة القوميات (عدد *= 18 *)، وإثيوبيا (عدد * = 19 *)، وغواتيمالا (عدد * = 20 *)، وجمهورية لاو الديمقراطية الشعبية (عدد * = 12 *)، وليبيريا (عدد * = 12 *)، ورواندا (عدد * = 25 *). تم تحديد مواقع المستشفيات باستخدام أسلوب أخذ العينات البراغماتي. وتم تشفير البيانات جغرافيًا ثم تحليلها باستخدام منصة للعرض للبصري للبيانات على الإنترنت. وقد تم تحديد كل فئة سكانية خاضعة للتغطية الخدمية للمستشفى حسب الأشخاص الذين يمكنهم الوصول إلى المستشفى من خلال رحلة بالمركبة لا تتعدى ساعتين. وتم اعتبار أن المستشفى لا تقدم التوافر المستمر للموارد الجراحية الأساسية إلا في حال التوفر الدائم للماء النظيف، والكهرباء، والأدوية الأساسية بما في ذلك السوائل الوريدية ومخدر واحد على الأقل، والمضاد الحيوي، ومسكن الألم، وجهاز عامل لقياس النبض، وجهاز عامل للتعقيم، والأكسجين، وأفراد الطاقم الطبي المعتمدين لإجراء العملية الجراحية والتخدير.

النتائج لم تنجح سوى 41 مستشفى (‏34.2‏%) من أصل 120 مستشفى خضعت الدراسة في استيفاء معايير توفير الخدمات الجراحية الأساسية المستمرة. اختلفت مناطق التغطية الخدمية المجمعة للمستشفيات المشمولة في الدراسة في كل دولة خضعت للدراسة بين 3.3 مليون شخص في ليبريا و151.3 مليون شخص في بنغلاديش. ولكن التجمعات المشتركة للمستشفيات الخاضعة للدراسة في كل دولة خضعت للدراسة ممن وافقت معايير تقديم الخدمات الجراحية الأساسية المستمرة كانت أصغر بصورة ملحوظة وتراوحت بين 1.3 مليون في ليبيريا و79.2 مليون في بنغلاديش.

الاستنتاج أظهرت العديد من المرافق الخاضعة للدراسة قصورًا في البنية التحتية الأساسية اللازمة لتوفير الرعاية الجراحية الأساسية بشكلٍ مستمر.

## 摘要

**目的:**

旨在评估七个国家选定机构持续提供基本外科资源的能力。

**方法:**

在 2010 年到 2014 年期间,我们使用情景分析工具收集了埃塞俄比亚 (*n* = 19)、多民族玻利维亚国 (*n* = 18)、老挝人民民主共和国 (*n* = 12)、利比里亚 (*n* = 12)、卢旺达 (*n* = 25)、孟加拉国 (*n* = 14) 以及危地马拉 (*n* = 20) 的地方和地区医疗机构的数据。我们通过务实的抽样方法选择医疗机构。然后使用一个在线数据可视化平台对数据进行地理编码和分析。我们将每家医院的服务范围人口定义为到达医院车程不超过 2 小时的人口。具有持续提供基本外科资源能力的医院被视为必须能够提供清洁用水、电力、必要的药物(包括静脉注射以及至少一种麻醉剂、止痛剂和抗生素)、功能齐全脉搏血氧计和灭菌剂、氧气以及执业外科手术医生和麻醉人员。

**结果:**

在调查的 120 家医院中,仅 41 (34.2%) 家医院满足标准,能够提供持续的基本外科服务。每个调查国家对象医院的综合服务范围人口在 330 万(利比里亚)和 15,130 万(孟加拉国)之间变动。但是,符合提供持续基本外科服务的调查国家对象医院的综合服务范围人口显著更小,在 130 万(利比里亚)和 7,920 万(孟加拉国)之间变动。

**结论:**

许多研究医疗机构在持续提供基本外科护理必要的基本设施方面存在不足。

## Резюме

**Цель:**

Оценить постоянную доступность основных хирургических ресурсов в отдельных медицинских учреждениях в семи странах.

**Методы:**

В 2010–2014 годах мы использовали инструмент ситуационного анализа для сбора данных в районных и областных больницах в Бангладеш (*n* = 14), Боливии (Многонациональное государство) (*n* = 18), Гватемале (*n* = 20), Лаосской Народно-Демократической Республике (*n* = 12), Либерии (*n* = 12), Руанде (*n* = 25) и Эфиопии (*n* = 19). Участки больниц были выбраны в условиях реальной клинической практики. Данные были геокодированы, а затем проанализированы с помощью онлайн-платформы визуализации данных. Популяция, обслуживаемая в каждой больнице, была определена как количество людей, которые могли бы добраться до больницы на автомобиле в течение не более двух часов. Больница рассматривалась только на предмет постоянной доступности основных хирургических ресурсов, т. е. были ли постоянно доступны чистая вода, электричество, необходимые лекарства, включая препараты для внутривенного введения и по меньшей мере один анестетик, анальгетик и антибиотик, функциональный пульсоксиметр, функциональный стерилизатор, кислород и медицинские работники, аккредитованные для проведения хирургических операций и анестезии.

**Результаты:**

Только 41 (34,2%) из 120 больниц, участвовавших в исследовании, отвечала критериям предоставления постоянных базовых хирургических услуг. Совокупные популяции, обслуживаемые исследуемыми больницами в каждой исследуемой стране, варьировались между 3,3 млн человек в Либерии и 151,3 млн человек в Бангладеш. Тем не менее совокупные популяции, обслуживаемые исследуемыми больницами в каждой исследуемой стране, которые соответствовали критериям предоставления постоянных базовых хирургических услуг, были существенно меньше и варьировались между 1,3 млн человек в Либерии и 79,2 млн человек в Бангладеш.

**Вывод:**

Многие из исследуемых медицинских учреждений испытывали недостаток в базовой инфраструктуре, необходимой для обеспечения базовой хирургической помощи на постоянной основе.

## Introduction

Access to emergency and essential surgery is integral to a comprehensive health-care system. Since the development of the millennium development goals, the global health community has increasingly recognized the role of surgical care in the treatment of common conditions such as acute abdominal processes, obstetric complications and trauma.^1^ Surgical conditions are estimated to account for 18% of the global burden of disease.^2^ However, in low- and middle-income countries there is often inadequate surgical capacity. In 2015, it was estimated that at least 143 million additional operations would be required to address emergency and essential surgical conditions in such countries.^3^ In the same year, the Lancet Commission on Global Surgery noted that 5 billion people did not have access to affordable, safe and/or timely surgical care^3^ and, each year, such lack of access results in an estimated 1.5 million avoidable deaths.^2^ The Lancet Commission also proposed six key indicators to define and measure the availability and affordability of surgical care for a given population^3^ – including case volume, the density of the surgical specialist workforce, perioperative mortality and timely access. Since 2011, several of these key indicators have been investigated.^4^^–^^8^

The impetus to understand and implement the basic components of the provision of quality surgical care is stronger than ever. With the recent implementation of the United Nation’s sustainable development agenda for 2030,^9^ there is renewed opportunity to focus on expanding universal health-care coverage to include essential surgical services. Moreover, to achieve sustainable development goal 3 – i.e. ensuring healthy lives and promoting well-being for all at all ages – a more detailed understanding of the calibre of the surgical care available in low- and middle-income countries is necessary. The substantial and often alarming variability observed in surgical mortality rates within and across countries^10^ supports the argument that surgery must occur within an appropriate framework that prioritizes the safety and welfare of patients.

The district hospital is expected to provide emergency and essential surgery and serve as the nexus of surgical services that do not require referral to specialized centres for tertiary care.^4^^–^^8^^,^^11^ While many district hospitals provide simple and essential surgical procedures, the resources and materials available to provide safe care are frequently inadequate. We decided to assess the difference in access to essential surgical services when minimum resource standards are included in the calculation of surgical availability. We used geographical information systems to investigate, in seven countries, the availability of basic surgical resources for patients who lived within a two-hour vehicle trip of one of a selection of hospitals that provided surgical services.

## Methods

In cooperation with health ministries or other partner institutions in each country, sample district or regional hospitals providing emergency and essential surgery were identified in Bangladesh, the Plurinational State of Bolivia, Ethiopia, Guatemala, the Lao People's Democratic Republic, Liberia and Rwanda. We selected these countries because they were considered relatively safe for researchers and offered apparently good opportunities for collaboration with local officials. The study hospitals were selected for convenience and proximity to national roadways. In each study country, unless access was limited by poor road conditions or safety concerns, at least one district hospital providing surgical services was assessed per county or district. If more than one hospital was accessible per county or district, we included all of them in our evaluation and categorized them as district hospitals or regional referral centres.

Between 2010 and 2014, each national survey was conducted by one of the study authors who, in collaboration with local health administrators, performed in-person interviews and on-site assessments of capacity to provide surgical and anaesthesia services. Hospital visits included face-to-face interviews with anaesthesiologists, hospital directors, nurses, pharmacists, physicians and surgeons. Medical directors provided permission for the researchers to tour relevant infrastructure, including the study hospitals’ pharmacies, operating rooms and wards. Audits were documented using an abbreviated version of the World Health Organization’s (WHO’s) Global Initiative for Emergency and Essential Surgical Care survey questionnaire.^7^^,^^12^^,^^13^ More detailed descriptions of this questionnaire are included in the reports of previous country-specific investigations.^4^^–^^8^

The Lancet Commission on Global Surgery proposed dimensions for access that included affordability, safety and timeliness.^3^ We could not assess affordability but assessed access – using a two-hour maximum travel time – and safety – using an on-site assessment of basic infrastructure.^3^ Through expert consensus, we identified a minimum set of eight resource criteria that, if met entirely by an individual facility, indicated that the facility was able to provide emergency surgical services consistently (Box 1). Consistency in this context meant that all interviewees at a study hospital reported that each of the eight resources assessed at their facility was “always available” rather than “available sometimes” or “never available”.

Box 1 **The eight resources considered essential for safe basic surgery at a hospital**

**Equipment and supplies**

- Consistent oxygen supply
- Essential medications – i.e. antibiotic, analgesic, inhaled or intravenous anaesthestic and intravenous fluids
- Functional pulse oximeter
- Functional sterilizer

**Infrastructure**

- Consistent electricity supply
- Consistent supply of clean water

**Personnel**

- Accredited anaesthesia provider
- Accredited surgical provider

Surgical facilities were geo-located using ArcGIS version 10.3 (ESRI, Redlands, United States of America) and analysed in Redivis (Redivis Inc., Mountain View, USA) – an online data visualization platform. Additional statistical analyses were performed in Stata version 11.0 (StataCorp. LP, College Station, USA). Estimates of catchment populations were based on the WorldPop database, which provides population densities in terms of individuals per square metre.^14^ Travel time to each hospital was estimated from the relevant road distances and estimated road speeds provided by OpenStreetMaps.^15^ For our analyses, we used so-called Manhattan distances – i.e. distances based on the road infrastructure – rather than Euclidean – i.e. straight-line distances. Following the Lancet Commission’s suggestion,^3^ we defined the catchment population of a study hospital as the number of people who could reach the hospital via a vehicle trip that lasted no longer than two hours. For each study country, we used geospatial techniques to map the discrepancy between the total catchment population of all the study hospitals and the catchment populations of the study hospitals that provided consistent emergency surgical services. We also assessed the proportions of the estimated national population in 2013^16^ represented by the catchment populations in each study country.

No patient data were collected and institutional review board exemption was obtained by partner institutions, as previously described.^4^^–^^8^^,^^17^

## Results

**Fig. 1:**
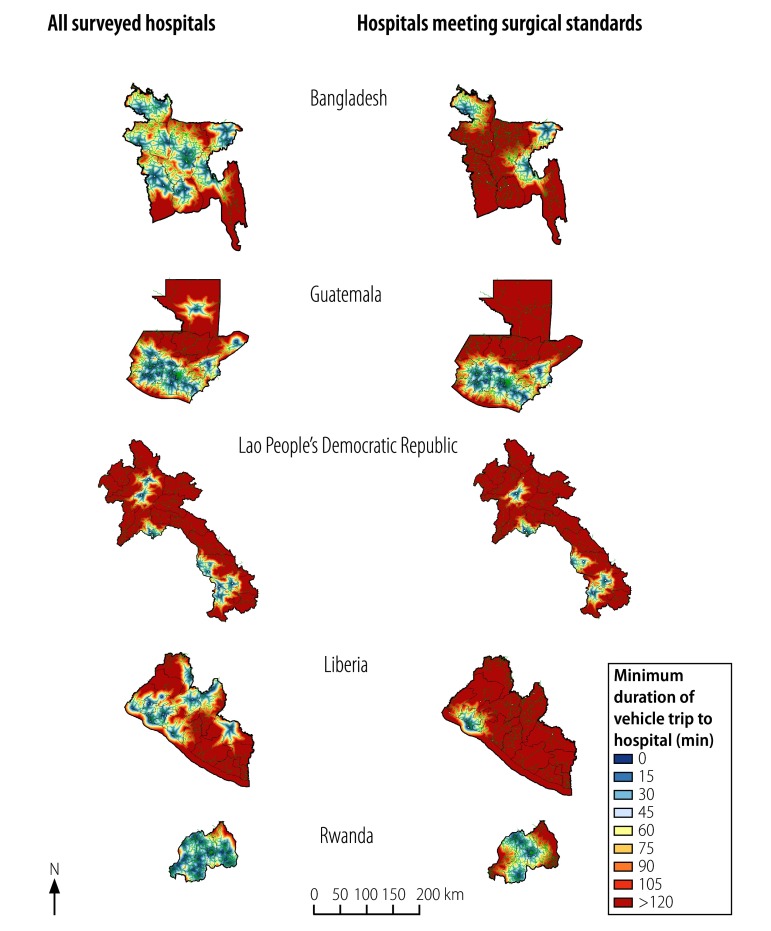
Estimated vehicle trip durations for attending any surveyed hospital or any surveyed hospital meeting basic surgical standards, Bangladesh, Guatemala, the Lao People's Democratic Republic, Liberia and Rwanda, 2010–2014

Notes: Areas shown in red were occupied by people who within 2 hours could probably not have reached a surveyed hospital (left column) or any surveyed hospital meeting basic surgical standards (right column). The estimated durations were based on road distances and estimates of typical vehicle speeds on the country’s main and secondary roads. The criteria for a hospital to be deemed meeting basic surgical standards are presented in Box 1. Roads are shown in green.

Source: Maps were generated through the computer program Redivis (Redivis Inc., Mountain View, USA), which uses OpenStreetMaps^15^ to populate its real-time maps.

**Fig. 2:**
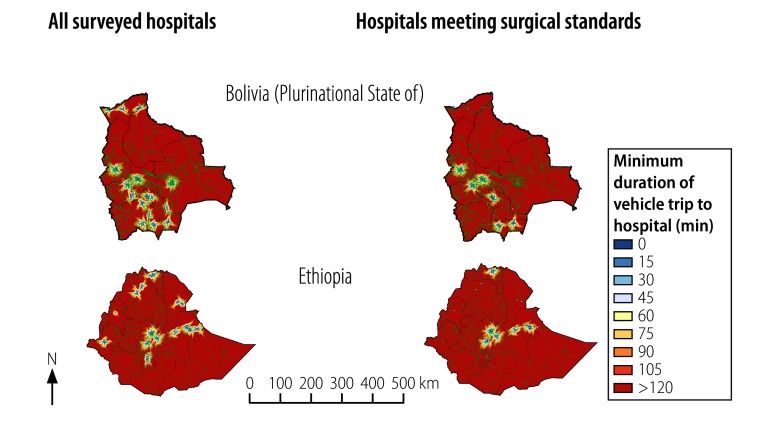
Estimated vehicle trip durations for attending any surveyed hospital or any surveyed hospital meeting basic surgical standards, the Plurinational State of Bolivia and Ethiopia, 2011

Notes: Areas shown in red were occupied by people who within 2 hours could probably not have reached a surveyed hospital (left column) or any surveyed hospital meeting basic surgical standards (right column). The estimated durations were based on road distances and estimates of typical vehicle speeds on the country’s main and secondary roads. The criteria for a hospital to be deemed meeting basic surgical standards are presented in Box 1. Roads are shown in green.

Source: Maps were generated through the computer program Redivis (Redivis Inc., Mountain View, USA), which uses OpenStreetMaps^15^ to populate its real-time maps.

Data were collected from a total of 120 hospitals identified as providing surgical care (Table 1). The estimated road travel time needed, by patients, to reach any of our surveyed hospitals – or any of our surveyed hospitals that met all eight resource criteria for basic surgery are illustrated in Fig. 1 and Fig. 2. The median size of a catchment population for a study hospital was 11.1 million (interquartile range, IQR: 3.6–34.8 million). The combined estimated catchment populations of the study hospitals in each country, which varied from 3.3 million people in Liberia and 151.3 million in Bangladesh, represented an estimated 37.0–99.9% of the national populations in 2013. The corresponding values for the 41 (34.2%) of the study hospitals that met all eight resource criteria for providing basic surgery consistently were substantially smaller. The combined catchment populations for such hospitals varied from 1.3 million in Liberia to 79.2 million in Bangladesh and represented an estimated 23.7–95.8% of the national populations in 2013 (Table 1). In each study country, the median number of individuals who lived in the catchments of study hospitals that appeared to be unable to provide basic surgery consistently was 2.0 million (IQR: 0.5–12.5 million; *P* = 0.014).

**Table 1:** Access to hospitals meeting basic surgical standards in terms of eight resource criteria, seven countries, 2010–2014

Country	Survey year	No. of hospitals		National population**^a^**	Combined catchment population (% of national population in 2013)**^b^**	
		Evaluated	Meeting BSS		All evaluated hospitals	Hospitals meeting BSS
Bangladesh	2012	14	3	156 600 000	151 275 600 (96.6)	79 239 600 (50.6)
Bolivia (Plurinational State of)	2011	18	9	10 670 000	8 141 200 (76.3)	5 548 400 (52.0)
Ethiopia	2011	19	7	94 100 000	34 817 000 (37.0)	22 301 700 (23.7)
Guatemala	2013	20	12	15 047 000	13 151 100 (87.4)	11 992 500 (79.7)
Lao People's Democratic Republic	2014	12	9	6 077 000	3 646 200 (60.0)	3 433 500 (56.5)
Liberia	2011	12	2	4 294 000	3 315 000 (77.2)	1 318 300 (30.7)
Rwanda	2010	25	5	11 078 000	11 066 900 (99.9)	10 612 700 (95.8)

BSS: basic surgical standards.

^a^ In 2013, according to the World Bank.^16^

^b^ Catchment populations represented the estimated number of people who, if using a road vehicle, could reach a study hospital within 2 hours. The estimated numbers were based on estimated vehicle speeds, population densities and typical conditions for each country’s main and secondary roads.

Note: The criteria for BSS are presented in Box 1.

### Bangladesh

In Bangladesh, we investigated 14 public hospitals. Seven of the study hospitals had affiliations with medical colleges and three of these college-affiliated hospitals were the only study hospitals in Bangladesh to meet the minimum resource criteria. Five of the study hospitals reported routine breaks in their electricity supplies.

### Plurinational State of Bolivia

Of the 18 hospitals surveyed in the Plurinational State of Bolivia, 11 were basic or district hospitals and seven general or referral hospitals. Only nine hospitals – three basic and six general – met all of the minimum criteria for providing basic surgery. Seven hospitals reported that they had a discontinuous water supply and seven reported that they had a discontinuous supply of electricity.

### Ethiopia

In Ethiopia, we collected data from 19 hospitals – six district and 13 regional. Only seven of the study hospitals– three district and four regional – met our basic surgical standards. The most common resource gaps were related to personnel and supplies: 12 of the study hospitals had no accredited surgical providers, nine had no accredited anaesthesia providers, seven had no functional pulse oximeters and six routinely experienced shortages in essential medications.

### Guatemala

One of the 20 hospitals surveyed in Guatemala was recognized as a referral centre. Only 12 of the study hospitals – including the referral centre – met our basic surgical standards. A lack of equipment and/or medications meant that the other eight study hospitals failed to meet all of the resource criteria. Most of the providers of anaesthesia and surgery we surveyed were primary-care physicians rather than specialists.

### Lao People's Democratic Republic

All 12 of the study hospitals surveyed in the Lao People's Democratic Republic appeared to have sufficient equipment for basic surgery but only nine of them – including all four in Vientiane prefecture or Vientiane province – had providers of anaesthesia and surgery and met all of our other basic surgical standards.

### Liberia

We recorded large disparities in surgical coverage across Liberia. Overall, 12 hospitals were assessed, one of which was a referral centre in the capital region of Montserrado. Only two of the study hospitals – one of which was the referral centre – met all of our basic surgical standards. Of the other 10 study hospitals, 10 and seven lacked consistent supplies of water and electricity, respectively, and nine reported routine shortages in essential medications. In most of the study hospitals, all oxygen was provided by a concentrator that was not dedicated to the operating theatre.

### Rwanda

The combined catchment population of the 21 district hospitals and four referral hospitals surveyed in Rwanda represented almost all (11.1 million people; 99.9%) of the estimated national population of 11.8 million people. Although only five of the 25 study hospitals – three district and two referral – met the basic surgical standards, the small size of the country meant that 10.6 million people – i.e. an estimated 95.8% of the national population – fell within the catchments of at least one of these hospitals. The other 20 study hospitals reported routine shortages of essential medications. Specialist surgeons and anaesthesiologists were concentrated in the referral hospitals and many general practitioners at district hospitals elected to send patients to the referral centres whenever possible.

## Discussion

We evaluated basic resources and infrastructure for emergency and essential surgical care at 120 hospitals in seven countries and noted that, despite all of these facilities providing surgical services, less than half met basic resource requirements. A median of about 2 million people in each study country lived in catchment areas of hospitals that appeared unable to provide reliable surgical services. In Bangladesh, 72.0 million people lived in such catchment areas. In the Lao People's Democratic Republic and Rwanda, the impact of the inconsistent availability of surgical services appeared less because the small geographical size of the country meant that most patients could reach a facility with basic surgical resources within two hours. Our results also showed that inconsistent availability of resources even affected referral centres. In some countries there is, potentially, a sizable proportion of patients who are being referred to these larger regional sites only to be met with a similar lack in resources.

The paucity of surgical resources in low-income settings – whether equipment-related, infrastructural or personnel-related – is an ongoing crisis requiring attention. Inconsistencies in resource availability affect the ability to provide timely, high quality surgical care. WHO has attempted to define the specific minimum requirements for surgical care through its Global Initiative for Emergency and Essential Surgical Care programme.^12^ Presumably, improved standards for surgical care would accompany improvements in infrastructure, qualified personnel and supplies. The ability to provide basic surgical services is dependent upon the simultaneous availability of multiple resources – coupled with strong management practices. Ample evidence exists that, in low- and middle-income countries, emergency and essential surgery is cost-effective and frequently needed.^18^^,^^19^

The introduction of essential medications lists was pivotal in changing the patterns of patient and provider access to life-saving drugs.^20^ Facilities providing emergency and essential surgery should have similar priority lists – of essential surgical provisions – that are supported by ministries and international organizations such as WHO. Such lists should lead to improved standards of patient monitoring – e.g. through the routine availability and use of pulse oximetry – and infection reduction – e.g. by improving access to antibiotics, clean water and sterilization processes. By establishing a list of the minimum surgical infrastructure, materials and other resources – and holding facilities and health systems accountable for the procurement and availability of the resources – the benchmark for surgical quality could be quickly raised. Although substantial investment would be required, it is likely that the improved delivery of surgical services would have a constructive impact on numerous hospital-wide services beyond surgical activities.

Our study has several limitations. The country-specific data constituted only a sampling of facilities and should not be considered truly representative of all surgical sites in the countries studied. However, within each study country, we did attempt to include at least one surgical facility per county or district at district-hospital level or higher. Feasibility constraints, safety concerns and time constraints meant that we did not visit – or even list – every surgical site in each country.

We used geographical mapping and estimates of road distances and mean vehicle speeds on roads with typical levels of congestion to delineate the population that could reach a study hospital, by road, within two hours. We ignored breakdowns in transportation, seasonal variation in road conditions, specific referral patterns between local hospitals and socioeconomic barriers to seeking care. Our underrepresentation of the population that did not have the means to travel in a road vehicle or, at least, without a long wait for a bus or other public transport – and, therefore, our overestimation of general access to surgical resources – seems likely. However, the mapping software we used was able to discriminate between main roads and secondary roads and to provide estimated road speeds based upon the probable congestion and quality of each type of road.

Data on surgical facilities are likely to become rapidly outdated: trained personnel relocate; unanticipated supply shortages occur; existing infrastructure may rapidly deteriorate; and new facilities may be built. Our data, which were collected over six years, are unlikely to reflect the current situation in any of our seven study countries. Most notably, the surgical system in Liberia was irrevocably altered by the effects of – and responses to – the 2013–2016 Ebola virus disease outbreak. A detailed, ongoing and regularly updated inventory of surgical facilities and resources in each country could be very useful.

We used geographical information systems to look at multiple hospitals providing surgery – as well as to examine the nuances in access to appropriate care as defined by basic surgical standards. If data collection were part of an ongoing evaluation process, such systems could help ministries of health target their efforts more effectively and evaluate improvements – or deterioration – over time.

In conclusion, the measurement of the quality of surgical care in resource-poor settings is a complex task. Analysis based on a set of minimum resource criteria for providing basic surgical care has emphasized the many gaps in surgical services in several resource-poor settings. In several of our study countries, many hospitals that, in theory, were providing surgical coverage to their catchment population were unable to meet basic surgical standards consistently. Many people in our study countries may have poor access to centres for emergency or essential surgical care and – because of resource constraints – the surgical care available to them may not be safe or of high quality.
